# Y Chromosome Microdeletions in Infertile Men with Non-obstructive Azoospermia and Severe Oligozoospermia

**Published:** 2017

**Authors:** Shin Young Kim, Hyun Jin Kim, Bom Yi Lee, So Yeon Park, Hyo Serk Lee, Ju Tae Seo

**Affiliations:** 1- Laboratory of Medical Genetics, Medical Research Institute, Cheil General Hospital and Women’s Healthcare Center, Seoul, Korea; 2- Department of Urology, Cheil General Hospital and Women’s Healthcare Center, Dankook University College of Medicine, Seoul, Korea

**Keywords:** Male infertility, Non-obstructive azoospermia, Reproductive hormone, Severe oligozoospermia, Y chromosome microdeletion

## Abstract

**Background:**

The purpose of the study was to investigate the frequencies and types of Y chromosome microdeletions in infertile men and to analyze the relationship between the levels of reproductive hormones and Y microdeletions.

**Methods:**

A total of 1,226 infertile men were screened for Y chromosome microdeletions using multiplex PCR assay. Karyotype analysis was performed on peripheral blood lymphocytes with standard G-banding. Serum reproductive hormone levels were measured.

**Results:**

Out of 1,226 infertile patients, 134 (10.93%) had Y microdeletions. One hundred seven of 765 (13.99%) non-obstructive azoospermic patients and 27 of 133 (20.30%) severe oligozoospermic patients had Y microdeletions. Among the 134 infertile men with Y microdeletions, the most frequent microdeletions were detected in the AZFc region, followed by AZFbc, AZFb, AZFa, AZFabc(Yq), Yp(SRY)+Yq, and partial AZFc regions. Karyotype analysis was available for 130 of the 134 patients with Y microdeletions. Of them, 36 (27.69%) patients had sex chromosomal abnormalities. Levels of FSH and LH in patients with AZFc microdeletion were significantly lower, while those in patients with Yp(SRY)+Yq were significantly higher than in patients without Y microdeletions. Level of testosterone in patients with AZFabc(Yq) or Yp(SRY)+Yq was significantly lower than that in patients without Y microdeletions. However, there was no significant difference in the levels of reproductive hormones between all patients with and without Y microdeletions.

**Conclusion:**

These results highlight the need for Y chromosome microdeletion screening for correct diagnosis of male infertility. Obtaining reliable genetic information for assisted reproductive techniques can prevent unnecessary treatment and vertical transmission of genetic defects to offspring.

## Introduction

Infertility is defined as failure to conceive after one year of unprotected sexual intercourse (1). This problem affects approximately 10%–15% of couples worldwide, and male-related factors are responsible for half of this case (2). Several factors have been implicated in male infertility such as hormonal abnormalities, erectile dysfunction, infections, antisperm antibodies, exposure to chemical agents and radiations, testicular cancer, varicocele, genetic factors, and others (3, 4). Thus, male infertility is a multifactorial syndrome encompassing a wide variety of disorders. However, in about 30%–50% of male cases, the etiology of infertility is still unknown.

Microdeletion of the azoospermia factor (AZF) region located on the long arm of the Y chromosome (Yq11) is considered the most common genetic cause of male infertility (5). The AZF region is divided into three nonoverlapping subregions called AZFa, AZFb, and AZFc, all of which are required for normal spermatogenesis. Microdeletions in these three regions are associated with various spermatogenetic alterations including Sertoli cell-only syndrome (SCOS), maturation arrest, and hypospermatogenesis. Specifically, microdeletion of AZFa is relevant to complete SCOS and azoospermia. The absence of AZFb is associated with maturation arrest at meiosis, whereas microdeletion of AZFc results in variable clinical and histologic phenotypes, ranging from oligozoospermia to SCOS (6). Extensive studies have been carried on Y microdeletions in non-obstructive azoospermic and severely oligozoospermic patients, with a reported incidence ranging from 3% to 28% (7, 8). Therefore, disruption of AZF can be viewed as the most common molecularly diagnosable cause of spermatogenic failure in the setting of non-obstructive azoospermia or severe oligozoospermia (9).

Recently, the techniques of testicular sperm extraction (TESE) and intracytoplasmic sperm injection (ICSI) have made it possible to help men with azoospermia or severe oligozoospermia to achieve successful fertilizations and pregnancies (10). However, Y microdeletions can be transmitted from infertile fathers to their male offspring, who could also experience infertility, through the procedure of ICSI. Thus, it is important to evaluate Y microdeletions in male infertility before assisted reproduction in order to provide appropriate information to patients.

The objective of this study was to investigate the frequencies and types of Y chromosome microdeletions by using multiplex polymerase chain reaction (PCR) in 1,226 infertile men. Moreover, the relationship between the levels of reproductive hormones and Y chromosome microdeletions was analyzed.

## Methods

### Subjects and semen analysis:

In this study, 1,226 infertile males were analyzed who were diagnosed and treated at the Department of Urology at Cheil General Hospital and Women’s Healthcare Center in Seoul, Korea, from April 2009 to August 2016.

All patients underwent physical examination, semen analysis, reproductive hormone estimation, karyotyping, and Y chromosome microdeletion analyses. Semen samples were obtained by masturbation into a sterile container after 3–5 days of sexual abstinence. Specimens were sent at room temperature to the laboratory and analyzed for sperm count, sperm volume, pH, motility, morphology, and fructose concentration was measured according to the guidelines of the World Health Organization (WHO) (11). All subjects underwent semen analysis at least twice. After semen analysis, patients suffering from azoospermia or severe oligozoospermia were offered the possibility of undergoing a testis biopsy to recover any spermatozoa suitable for ICSI and microsurgical multiple TESE. A structured questionnaire was used to collect information about each subject’s medical history and demographic characteristics. Appropriate institutional review board approval was obtained from the Ethics Committee at Cheil General Hospital and Women’s Healthcare Center for this study (#CGH-IRB-2016-46). Written informed consent was obtained from each participant before the collection of samples and subsequent analyses.

### Molecular analysis:

Peripheral blood was collected in EDTA vacutainer tubes (Becton Dickinson, USA), and genomic DNA was extracted from whole blood using a QIAamp DNA Blood Mini Kit (Qiagen GmbH, Germany). Y chromosome microdeletions were detected using multiplex PCR amplification with specific sequence-tagged sites (STS) using 16 sets of primers. This allowed evaluation of the following sites: sY14 (sex-determining region Y, SRY gene) and ZFY (X-linked gene encoding a zinc-finger protein) for internal control regions; sY84 and sY86 for the AZFa region; sY124, sY127, sY129, sY130, and sY134 for the AZFb region; sY147, sY242, sY254, sY255, SPGY1, sY157, and sY158 for the AZFc region (Supplementary table 1). STS multiplex PCR sets and their amplified fragments are shown in supplementary table 1. PCR was carried out in 10 *μl* reaction volumes containing 50 *ng* of genomic DNA, 1×PCR buffer, 1.5 *mM* MgCl_2,_ 1 *mM* of each dNTP, 10 *pmol* of each specific primer, and 1 unit of AmpliTaq Gold DNA polymerase (Thermo Fisher, USA). The PCR conditions consisted of an initial denaturation at 95°*C* for 10 *min*, followed by 35 cycles of denaturation at 95°*C* for 30 *s*, annealing at 62°*C* for 1 *min* 30 *s*, extension at 65°*C* for 1 *min* 30 *s*, and a final extension at 65°*C* for 10 *min* on an ABI PRISM 2700 thermal cycler (Thermo Fisher). Amplification products were separated by electrophoresis on 3% NuSieve gels containing ethidium bromide (0.1 *mg/ml*) and were visualized under ultraviolet light. In each PCR reaction, normal female and fertile male DNA samples were used as negative and positive controls, respectively. Water was used as a blank control to check for any DNA contamination. All samples were analyzed in a blinded fashion, without knowledge of the patient’s clinical details. Each experiment was performed at least twice.

**Table 1.** Comparison of clinical outcomes and characteristics in infertile men with and without Y chromosome microdeletion

	**Total**	**No microdeletion**	**Microdeletion**							

			**AZFa**	**AZFb**	**AZFc**	**AZFbc**	**AZFabc (Yq)**	**Yp(SRY)+Yq**	**Partial AZFc**	**Total**
**No. of patients**	1,226	1,092	10	11	69	28	7	5	4	134
**Age (y)**	37.57±4.90	37.63±4.97	36.60±4.43	37.18±3.16	36.42±4.05^*^	37.54±5.51	35.29±3.45	40.80±2.68	39.75±2.22	36.93±4.32
**Testis volume (*ml*)**										
Right	13.91±6.71	13.96±6.77	11.5±2.68	19.27±6.05^*^	14.33±4.66	12.11±5.01	6.71±4.82^*^	2.00±0.00^*^	20.67±9.02	13.32±5.88
Left	13.75±6.71	13.80±6.50	11.20±2.39	17.91±5.92^*^	13.94±4.42	14.18±13.77	5.86±4.22^*^	2.20±0.45^*^	15.50±4.12	13.28±8.00
**Semen analysis**										
Volume (*ml*)	2.58±1.57	2.56±1.58	2.31±1.20	3.75±2.39^*^	3.00±1.37^*^	2.72±1.51	1.81±1.51	1.21±0.82	2.68±1.46	2.80±1.55
pH	7.22±1.00	7.20±1.03	7.07±1.17	7.44±0.29	7.39±0.32	7.11±1.31	7.54±0.22	7.38±0.53	7.60±0.00	7.33±0.72
**Sperm concentration (× 10^6^/*ml*)**										
0	1,001	894	10	11	43	28	7	5	3	107
<5	140	113	0	0	26	0	0	0	1	27
≥5 and <14	48	48	0	0	0	0	0	0	0	0
≥14	37	37	0	0	0	0	0	0	0	0
TESE	623	559	5	7	37	12	1	1	1	64
**Reproductive hormones**										
FSH (*mIU/ml*)	18.09±14.69	18.26±15.13	20.72±8.60	11.04±7.39	14.35±8.81^*^	17.23±9.01	26.07±13.84	32.60±17.41^*^	18.37±9.98	16.65±10.34
LH (*mIU/ml*)	8.36±6.60	8.45±6.76	7.12±3.91	6.81±3.28	6.20±3.29^*^	8.39±4.41	12.17±8.24	19.60±9.17^*^	5.37±1.09	7.61±5.03
T (*ng/ml*)	3.87±1.89	3.88±1.90	3.65±1.92	4.16±1.39	4.30±1.98	3.37±1.54	2.17±1.58^*^	1.39±1.02^*^	3.33±0.26	3.78±1.90
PRL (*ng/ml*)	9.00±5.87	8.90±5.86	12.28±7.68	9.06±2.63	9.61±4.95	10.64±8.75	9.99±3.27	7.70±4.27	6.08±2.82	9.82±5.96
E_2_ (*pg/ml*)	20.36±10.16	20.52±10.15	21.80±9.85	18.12±9.62	19.07±9.28	22.02±12.54	9.79±8.03^*^	17.43±7.56	11.64±4.42	19.04±10.20

AZF, azoospermia factor; TESE, testicular sperm extraction; FSH, follicle stimulating hormone; LH, luteinizing hormone; T, testosterone; PRL, prolactin; E_2_, estradiol.

Comparisons between outcome groups were by Student’s t-test for continuous variables and Chi-square test or Fisher’s exact test for categorical variables.

* p<0.05, significant difference compared to infertile patients without Y chromosome microdeletions

**Supplementary table 1.** Sequence-tagged sites and gene-specific primer sequences for Y chromosome microdeletion analysis

**Multiplex PCR set**	**STS**	**Locus**	**Region**	**Sequence 5’→3’**		**Size (*bp*)**
**STS-1**		ZFX	X	Forward Reverse	CCATTCACACGAAAGACTATCC AGACCTGACTGTAAAATCTCCC	585
	sY14	SRY	Yp	Forward Reverse	GAATATTCCCGCTCTCCGG GCTGGTGCTCCATTCTTGAG	470
	sY254	DAZ	AZFc	Forward Reverse	GGGTGTTACCAGAAGGCAAAATC GAACCGTATCTACCAAAGCAGC	380
	sY86	DYS273	AZFa	Forward Reverse	GTGACACACAGACTATGCTTC ACACACAGAGGGACAACCC	318
	sY127	DYS218	AZFb	Forward Reverse	CTAGGCTCACAAACGAAAAG CTGCAGGCAGTAATAAGGG	277
**STS-2**		ZFX	X	Forward Reverse	CCATTCACACGAAAGACTATCC AGACCTGACTGTAAAATCTCCC	585
	sY14	SRY	Yp	Forward Reverse	GAATATTCCCGCTCTCCGG GCTGGTGCTCCATTCTTGAG	470
	sY134	DYS224	AZFb	Forward Reverse	GCTTAAAATGTTTGAGAAGCC CATCATGCTATGCACTTCAG	249
	sY255	DAZ	AZFc	Forward Reverse	GTTACAGGATTCGGCGTG CTCGTCATGTGCAGCCAC	123
**STS-3**		ZFX	X	Forward Reverse	CCATTCACACGAAAGACTATCC AGACCTGACTGTAAAATCTCCC	585
	SPGY1	DAZ	AZFc	Forward Reverse	ACAATTTTGATAGTCTGAACACAAGC TTCACATACAGCCATTAAGTTTAGC	456
	sY158	DYS241	AZFc	Forward Reverse	CTCAGAAGTCCTCCTAATAGTTCC CAGTGGTTTGTAGCGGGTAG	230
	sY129	DYS220	AZFb	Forward Reverse	AGCTTCAGGAGGTTCAAAAC AAGTGGGACCTAAGCTACG	194
	sY147	DYF83S1	AZFc	Forward Reverse	TTTCTCGTTTGATGATCCTAG TTAATATGAGAATGAGAACAGATG	100
**STS-4**		ZFX	X	Forward Reverse	CCATTCACACGAAAGACTATCC AGACCTGACTGTAAAATCTCCC	585
	sY84	DYS148	AZFa	Forward Reverse	AGAAGGGTCTGAAAGCAGG GCCTACTACCTGGAGGCTTC	326
	sY157	DYS240	AZFc	Forward Reverse	CTTAGGAAAAAGTGAAGCCG CCTGCTGTCAGCAAGATACAG	286
	sY242	DAZ	AZFc	Forward Reverse	ACACAGTAGCAGCGGGAGTTAC TCTGCCACTAAACTGTAAGCTCC	233
	sY130	DYS221	AZFb	Forward Reverse	AGAGAGTTTTCTAACAGGGCG TGGGAATCACTTTTGCAAC	173
	sY124	DYS215	AZFb	Forward Reverse	CAGGCAGGACAGCTTAAAAG ACTGTGGCAAAGTTGCTTTC	109

STS, sequence-tagged sites; SRY, sex-determining region Y; ZFX, zinc finger protein, X-linked; AZF, azoospermia factor

### Karyotype analyses:

Peripheral blood was collected in sodium heparin vacutainer tubes (Becton Dickinson, USA). Lymphocytes were cultured in RPMI 1640 medium (Welgene, Daegu, Korea) at 37°*C* for 72 *hr*. Karyotype analysis was performed on metaphase spreads of cultured peripheral lymphocytes. Karyotypes were analyzed using standard GTL- and RBG-banding techniques. CBG-, DA-DAPI- and NOR-staining confirmed the location of the centromere and the heterochromatic region of the Y chromosome. At least, 50 metaphases were analyzed for each patient and up to 100 metaphases in cases of mosaicism at the high resolution level of the 700-band per haploid chromosome set.

### Hormone analysis:

Peripheral blood was collected in a vacutainer serum separator tube (Becton Dickinson, USA). Levels of serum follicle-stimulating hormone (FSH), luteinizing hormone (LH), testosterone (T), prolactin (PRL), and estradiol (E_2_) were measured using chemiluminescence immunoassay and radioimmunoassay. Normal male reference ranges were FSH, 1.5–12.4 *mIU/ml*; LH, 1.7–8.6 *mIU/ml*; T, 2.41–8.27 *ng/ml*, PRL, 4.04–15.2 *ng/ml*, and E_2_, 7.4–42.6 *pg/ml*.

### Statistical analysis:

Data are expressed as mean ± standard deviation (SD) or number (%). Comparisons between outcome groups were performed using Student’s t-test for continuous variables and Chi-square test or Fisher’s exact test for categorical variables. Statistical significance was considered when p≤0.05. Statistical analysis was carried out using the Statistical Package for Social Sciences version 12.0 (SPSS Inc., Chicago, IL, USA).

## Results

**Figure 1.**
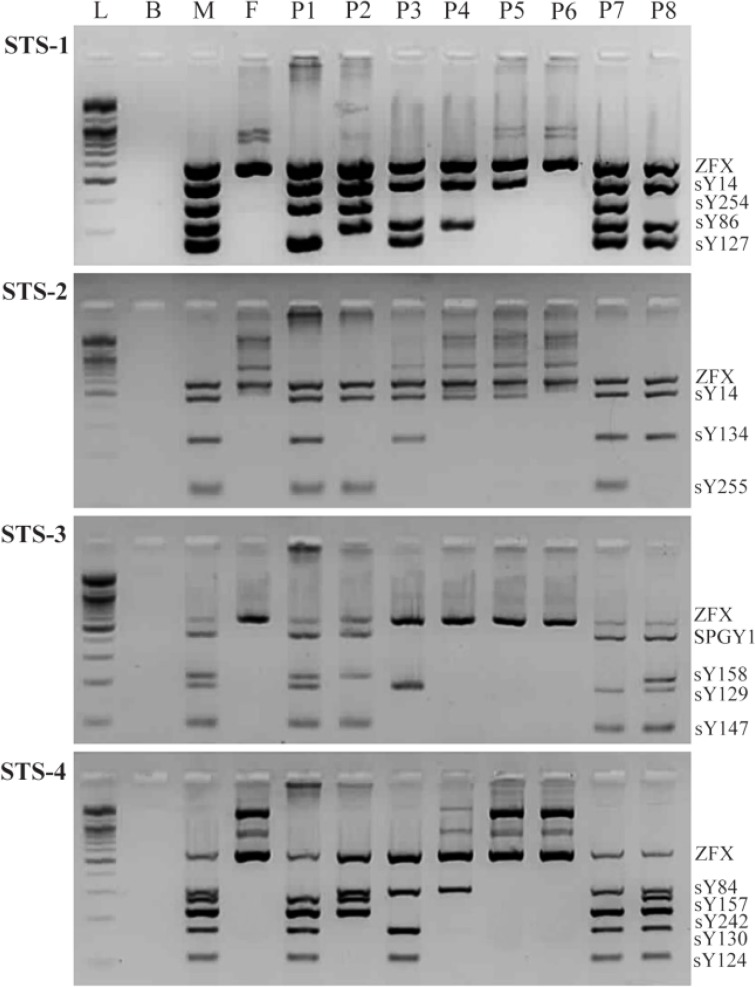
The microdeletion types of the azoospermia factor (AZF) regions in the Y chromosome by multiplex PCR analysis with STS markers. L: 100 *bp* DNA ladder; B: blank; M: male positive control, F: female negative control, P1: AZFa deleted patient; P2: AZFb deleted patient; P3: AZFc deleted patient; P4: AZFbc deleted patient; P5: AZFabc (Yq) deleted patient; P6: Yp(SRY)+Yq deleted patient; P7: partial AZFc (sY157 and sY158) deleted patient; P8: partial AZFc (sY254 and sY255) deleted patient

This study performed molecular screening for Y chromosome microdeletions in 1,226 infertile patients. The overall frequency of Y chromosome microdeletions was 10.93% (134/1,226). Clinical data of patients with Y chromosome microdeletions are summarized in table 1. Of the 134 patients with Y chromosome microdeletions, deletion of the AZFc region was the most frequent, 51.49% (69/134), followed by the AZFbc, 20.90% (28/134), AZFb, 8.21% (11/134), AZFa, 7.46% (10/134), AZFabc(Yq), 5.22% (7/134), Yp(SRY) +Yq, 3.73% (5/134), and partial AZFc, 2.99% (4/134) regions. The types of patients with these deletions are shown in figure 1. The mean age of infertile patients with and without Y chromosome microdeletions was 36.93±4.32 years (range: 27–53 years) and 37.63±4.97 years (range: 21–65 years), respectively. Testicular volumes on both sides were reduced in infertile patients with AZFabc(Yq) or Yp(SRY)+Yq deletion compared to infertile patients without Y microdeletion, whereas testicular volumes were increased in infertile patients with AZFb deletion (normal reference: >15 *ml*). However, there was no significant difference in testicular volumes between total infertile patients with and without Y microdeletions. As expected, sperm concentration was significantly lower in infertile patients with Y microdeletion than in infertile patients without Y microdeletion. Sixty-four (47.76%) of the 134 infertile men with Y chromosome microdeletions underwent microsurgical multiple TESE.

As shown in table 2, 107 of the 765 non-obstructive azoospermic (13.99%) (usually referred to as “no sperm count”) and 27 of the 133 severely oligozoospermic (20.30%) patients (sperm count <5×10^6^/*ml*) had a Y chromosome microdeletion. The most frequent deletions were detected in the AZFc region, which was seen in 43 non-obstructive azoospermic (43/765, 5.62%) and 26 severe oligozoospermic men (26/133, 19.55%). Deletions in the AZFa (10/765, 1.31%) or AZFb (11/765, 1.44%) region were only found in non-obstructive azoospermic patients. Moreover, large deletions involving the AZFbc, AZFabc (Yq), and Yp (SRY)+Yq regions were detected in 28 (28/133, 3.66%), seven (7/133, 0.92%), and five (5/133, 0.65%) non-obstructive azoospermic patients, respectively. Three non-obstructive azoospermic and one severe oligozoospermic patients had partial AZFc microdeletion. However, there was no significant difference in Y chromosome microdeletion frequency between the two groups (p= 0.079). Histologic diagnosis identified one case of SCOS, 38 of maturation arrest (MA), and five of hypospermatogenesis (HS) among infertile patients with Y microdeletion.

**Table 2.** The frequencies and types of Y chromosome microdeletions in infertile patients with non-obstructive azoospermia or severe oligozoospermia

	**Non-obstructive azoospermia (n=765) (%)**	**Severe oligozoospmia (n=133) (%)**	**Total, n (%)**	**p-value**	**Testicular histology**			

					**SCOS**	**MA**	**HS**	**NA**
**AZFa**	10 (1.31)	0 (0.0)	10 (7.46)	0.374	0	5	0	5
**AZFb**	11 (1.44)	0 (0.0)	11 (8.21)	0.384	0	6		5
**AZFc**	43 (5.62)	26 (19.55)	69 (51.49)	<0.001^*^	1	11	4	53
**AZFbc**	28 (3.66)	0 (0.0)	28 (20.90)	0.015^*^	0	14	0	14
**AZFabc(Yq)**	7 (0.92)	0 (0.0)	7 (5.22)	0.602	0	1	0	6
**Yp(SRY)+Yq**	5 (0.65)	0 (0.0)	5 (3.73)	1.000	0	1	0	4
**Partial AZFc**	3 (0.39)	1 (0.75)	4 (2.99)	0.474	0	0	1	3
**Total deletions, n (%)**	107 (13.99)	27 (20.30)	134	0.079				

SCOS, sertoli cell only syndrome; MA, maturation arrest; HS, hypospermatogenesis; NA, not available

Severe oligozoospermia: <5×10^6^ *sperm/ml*

Comparison of severe oligozoospermic group with non-obstructive azoospermic group by Fisher’s exact test

* p<0.05, significant difference compared to infertile patients with severe oligozoospermia

Karyotype analysis was available for 1,205 (98.29%) of the 1,226 infertile men. The overall frequency of chromosomal abnormalities was 16.93% (204/1,205). Among the 134 infertile men with Y microdeletion, karyotype analysis was available for 130 (97.02%). Thirty-six (all with non-obstructive azoospermia) of 130 patients (27.69%) had chromosomal abnormalities. The results and details of the Y microdeletion patients’ karyotypes are summarized in table 3. Abnormal karyotypes among these 36 patients included

1- 46,X,del(Y) (n=4);
2- 46,X,del(Y)/45,X,del(Y),der(21;22) (n=1);
3- 46,X,idic(Y) (n=8);
4- 46,X,idic(Y)/45,X (n=5);
5- 46,X,idic(Y)/45,X/46,X,+mar (n=1);
6- 46,XX (n=8);
7- 46,XX,t(6;20) (n=1);
8- 46,XX/45,X/46,X,der(Y) (n=1);
9- 46,X, mar/45,X (n=2); 46,X,der(X)t(X;?) (n=1);
10- 46,X,der(Y) (n=1);
11- 45,X/47,X,idic(Y),idic(Y) (n=1);
12- 45,X,i(Y)/45,X (n=1);
13- 45,X,t(2;20)/46,X,idic(Y)t(2;20) (n=1),

all of which are related to sex chromosome abnormality. It was revealed that 10 infertile patients with AZFa deletion and 10 with AZFb deletion, with one exception, had a 46,XY karyotype. Infertile patients with AZFabc(Yq) or Yp(SRY)+Yq deletion all had abnormal karyotypes. Table 1 compares the hormone levels of infertile groups with Y microdeletions with those of infertile patients without Y microdeletions. Levels of FSH and LH in infertile patients with Yp(SRY)+Yp deletions were significantly higher than those in infertile patients without Y microdeletions (p<0.05), whereas those in infertile patients with AZFc deletion were significantly lower (p<0.05). Level of T in both infertile patients with AZFabc(Yq) and Yp(SRY)+Yq microdeletions was significantly lower than that in infertile patients without Y microdeletions (p<0.05 for both). Level of E_2_ in infertile patients with AZFabc(Yq) microdeletion was significantly lower than that in infertile patients without Y microdeletions (p<0.05). However, levels of these hormones in all patients with Y microdeletions were not significantly different from infertile patients without Y microdeletions. Of 46,XX males cases (also known as testicular disorders of sex development), 46,XX males with Yp(SRY)+(Yq) deletion increased levels of FSH and LH, and decreased levels of T. In cases of 46,XX males with Yq deletion, FSH level was increased, whereas E_2_ level was decreased. All 46,XX male patients showed small testes with azoospermia, and height of patients ranged from 156 to 172 *cm*. There were no cases of ovo-testicular disorders of sex development or ambiguous genitalia.

**Table 3.** Overview of all patients karyotype details according to the nature of the Y microdeletion in 134 Y deleted inferile men

Deletion site	Total n (%) of patients	Kind of karyotype			Abnormal karyotype

		NA	Normal	Abnormal	
**AZFa**	10 (7.46)	0	10	0	----

**AZFb**	11 (8.21)	0	10	1	46,X,del(Y)(q11.23)

**AZFc**	69 (51.49)	3	63	3	46,X,del(Y)(q11.223)[80]/45,X,del(Y),der(21;22)(q10;q10)[60] (n=1)
					46,X,del(Y)(q11.23) (n=1)
					46,X,idic(Y)(q11.223) (n=1)

**AZFbc**	28 (20.90)	1	9	18	46,X,del(Y)(q11.223) (n=1)
					46,X,idic(Y)(q11.221) (n=1)
					46,X,idic(Y)(q11.221)[93]/45,X[7] (n=1)
					46,X,idic(Y)(q11.222) (n=5)
					46,X,idic(Y)(q11.222)[95]/45,X[14] (n=1)
					46,X,idic(Y)(q11.222)[49]/45,X[2] (n=1)
					46,X,idic(Y)(q11.222)[67]/45,X[33] (n=1)
					46,X,idic(Y)(q11.222)[94]/45,X[6] (n=1)
					46,X,idic(Y)(q11.222)[98]/45,X[39]/46,X,+mar[13] (n=1)
					46,X,idic(Y)(q11.223) (n=1)
					45,X[93]/47,X,idic(Y)(q11.23),idic(Y)[13] (n=1)
					46,XX[50]/45,X[25]/46,X,der(Y)(pter->q11.23::p11.2->pter)[25] (n=1)
					46,X,+mar[79]/45,X[21] (n=1)
					46,X,+mar[90]/45,X[10] (n=1)

**AZFabc(Yq)**	7 (5.22)	0	0	7	46,XX (n=3)
					46,XX[50] (n=1)
					46,X,del(Y)(q11.?21q11.?23) (n=1)
					46,X,der(X)t(X;?)(p22.33;?) (n=1)
					45,X,i(Y)(p10)[84]/45,X[18] (n=1)

**Yp(SRY)+Yq**	5 (3.73)	0	0	5	46,XX (n=4)
					46,XX,t(6;20)(q13;p12.2) (n=1)

**Partial AZFc (157, 158)**	3 (2.24)	0	1	2	45,X,t(2:20)(p10:p10)[135]/46,X,idic(Y)(q11.23)t(2:20)[51] (n=1)
					46,X,der(Y)(pter->q11.23::p11.2->pter) (n=1)

**Partial AZFc (254, 255)**	1 (0.75)	0	1	0	----

**Total n**	134	4	94	36	----

NA: not available

## Discussion

The microdeletions in the AZF region of the Y chromosome were investigated in 1,226 infertile patients from Korea. The overall frequency of AZF microdeletions was 10.93% (134/1,226). The frequency of microdeletions (10.93%) detected in the present study is within the range reported in previous studies 5.7% to 21.0% worldwide and 9.6% to 19.4% in Asian populations (12, 13). This result was slightly higher than that reported by Park et al. (2013) (14) in Korea (8.75%, 168/1,919). Similar to our results, the incidence of AZF microdeletions in 1,333 infertile patients from China was 10.80% (15). Zhu et al. (2008) (16) reported that the prevalence of AZF microdeletions on the Y chromosome of Chinese infertile men as detected by multi-analyte suspension array technology was 11.5%. In addition, the frequency of Y microdeletions was found at a rate of 13.99% (107/765) and 20.30% (27/133) in non-obstructive azoospermic and severely oligozoospermic patients, respectively. These results are similar to the published data of 11.75% in azoospermic patients and 18.8% in severely oligozoospermic patients (15, 17). However, literature reviews have uncovered a high frequency (51.6%) of microdeletions among azoospermic patients and a low frequency (less than 5%) in men with severe oligozoospermia (18, 19). This discrepancy and wide variation in deletion frequency estimates might be due to ethnic differences, sample sizes, patient selection criteria, methodological aspects, and even the type and number of markers used in the studies.

In the present study, AZFc deletion was the most frequent (51.49%), followed by AZFbc (20.90%), AZFb (8.21%), AZFa (7.46%), AZFabc(Yq) (5.22%), Yp(SRY)+Yq (3.73%), and partial AZFc (2.99%) deletions. Our finding is partially similar to a relevant previous Korean study that revealed a high frequency of microdeletions in the AZFc (56.55%) as compared to deletions in AZFa, 7.74%, AZFb, 5.95%, AZFbc, 22.02%, and AZFabc, 7.74% (14). However, Yp(SRY)+Yq and partial AZFc deletions were not found in their azoospermic and severely oligozoospermic patients (14). The AZFc deletion was the most common pattern of AZF microdeletions in patients with non-obstructive azoospermia and severe oligozoospermia; this finding is consistent with previous reports (9). Several candidate fertility genes have been discovered within the AZFc region. It is still not clear why AZFc deletion is so frequent, but it could be caused by repetitive sequences in this region. It has been suggested that men with AZFc deletion are capable of producing sperm, but some patients do not have any sperm inside their seminiferous tubules (20). In previous study, patients with AZFc deletion have shown a good prognosis for successful retrieval of sperm by TESE, whereas patients with deletions in the AZFa and AZFb regions have not (1). These findings are in agreement with those from this study, where AZFa, AZFb, AZFbc, AZFabc(Yq), and Yp(SRY)+Yq deletions were found only in non-obstructive azoospermic males. Partial AZFc deletion (sY157/s Y158 or sY254/sY255) was found in three non-obstructive azoospermic patients and one severe oligozoospermic patient in our study. Previous study has suggested a link between a partial deletion in the AZFc region and spermatogenic failure (21). However, other researchers have disagreed, proposing that this is simply a polymorphic deletion with no clinical ramifications (22).

Several previous studies have reported a range (2%–16%) of chromosomal abnormalities in infertile patients (23, 24). The total frequency (16.93%) of chromosomal abnormalities found in this study is within the range of the published data. The frequency of chromosomal abnormalities in our patients with Y chromosome microdeletions was 27.69%. In addition, large deletions, including those in the AZFbc, AZFabc(Yq), and Yp(SRY)+ Yq regions, can cause chromosomal instability and can be responsible for chromosomal rearrangements or Y chromosome loss. In our study, the frequency of chromosomal abnormalities was 66.67% (18/27), 100% (7/7), and 100% (5/5) in the AZFbc, AZFabc(Yq), and Yp(SRY)+Yq regions, respectively. Moreover, all of these abnormalities involved the sex chromosome, with a majority of 46,X,idic(Y), 46,X,idic(Y)/45,X, 46,XX, or 46,X,del(Y) abnormalities. An association between Y chromosome microdeletions and an isodicentric Y chromosome or 46,X,idic(Y)/45,X chromosomal mosaicism has been proposed previously (25, 26). These results suggest that genes in the AZF region are not only associated with spermatogenesis, but also with the stability and viability of the Y chromosome, such that microdeletions are an intrinsic element of AZF gene polymorphisms that can lead to translocations and deletions, together with gain or loss of Y chromosomes.

Levels of FSH and LH in infertile patients with Yp(SRY)+Yq deletions were significantly higher, whereas those in patients with AZFc deletion were significantly lower than those in infertile patient without Y microdeletions. The level of T in patients with AZFabc(Yq) or Yp(SRY)+Yq deletions was significantly lower than that in patients without microdeletions. However, there were no significant differences in the levels of FSH, LH, T, PRL, or E_2_ between total patients with and without Y microdeletions. This is in agreement with the results of a previous study (27). In contrast, the levels of FSH and T in patients with Y microdeletions were significantly lower than that in patients without Y microdeletions, whereas the level of LH was significantly higher in an Indian study (28). Surveys have also shown changes in male hormone physiology according to levels of FSH, LH, and T as well as modification of gonadal morphology in individuals affected by microdeletions of the Y chromosome (7, 28). Further research is necessary to determine the nature of the correlation between Y microdeletions in infertile patients and levels of these reproductive hormones.

More and more infertile men are choosing assisted reproduction techniques, such as TESE and ICSI, to have offspring. With the help of assisted reproduction techniques, it is possible for patients with severe impaired spermatogenesis to father children. However, these technologies can increase the risk of transmitting genetic disorders. Thus, before undergoing assisted reproduction, an understanding of the genetic defects responsible for infertility is essential to avoid unnecessary treatments and vertical transmission of these abnormalities to the offspring.

## Conclusion

This study demonstrated that Y chromosome microdeletion is a major genetic cause of primary male azoospermia. Detection of Y chromosome microdeletions is of great use for guiding clinical diagnosis, helping selecting treatment schemes, and reducing the incidence of genetic diseases. In this study, the importance of Y chromosome microdeletion screening and genetic counseling is strongly emphasized for infertile men prior to employment of assisted reproduction techniques.
